# Singing Experience Influences RSST Scores

**DOI:** 10.3390/healthcare10020377

**Published:** 2022-02-16

**Authors:** Naomi Yagi, Yoshitada Sakai, Naoko Kawamura, Hitoshi Maezawa, Yutaka Hata, Masayuki Hirata, Hideki Kashioka, Toshio Yanagida

**Affiliations:** 1 Faculty of Health Care Science, Himeji Dokkyo University, Himeji 670-8524, Japan; naomi@gm.himeji-du.ac.jp (N.Y.); kawamura@gm.himeji-du.ac.jp (N.K.); 2 Division of Rehabilitation Medicine, Kobe University Graduate School of Medicine, Kobe 650-0017, Japan; 3 Department of Neurological Diagnosis and Restoration, Graduate School of Medicine, Osaka University, Suita 565-0871, Japan; maezawa@ndr.med.osaka-u.ac.jp (H.M.); mhirata@ndr.med.osaka-u.ac.jp (M.H.); 4 Graduate School of Information Science, University of Hyogo, Kobe 650-0047, Japan; hata@gsis.u-hyogo.ac.jp; 5 Center for Information and Neural Networks, National Institute of Information and Communications Technology, Suita 565-0871, Japan; hideki.kashioka@nict.go.jp (H.K.); yanagida@nict.go.jp (T.Y.)

**Keywords:** singing, voluntary swallowing, RSST, inducibility of swallowing flex

## Abstract

It has recently been shown that the aging population is refractory to the maintenance of swallowing function, which can seriously affect quality of life. Singing and vocal training contribute to mastication, swallowing and respiratory function. Previous studies have shown that singers have better vocal cord health. No consensus has been reached as to how vocal training affects swallowing ability. Our study was designed to establish evidence that singers are statistically superior at inducing the swallowing reflex. To test our hypothesis, we undertook a clinical trial on 55 singers and 141 non-singers (mean age: 60.1 ± 11.7 years). This cross-sectional study with propensity score matching resulted in significant differences in a repetitive saliva swallowing test among singers: 7.1 ± 2.4, *n* = 53 vs. non-singers: 5.9 ± 1.9, *n* = 53, *p* < 0.05. We conclude that singing can serve an important role in stabilizing the impact of voluntary swallowing on speech.

## 1. Introduction

Pneumonia, including aspiration pneumonia, is a common cause of death in the elderly population [1,2]. The World Health Organization has noted that dysphagia leads to an increased risk of mortality. The likelihood of dysphagia increases with age, and substantially affects the morbidity of older adults. Video fluoroscopy and endoscopy, which are clinically performed to obtain accurate findings, are considered the gold standards for evaluating dysphagia. As these invasive diagnostic techniques cannot be used frequently, screening tests are often performed and noninvasive methods have been developed [3,4,5]. One of widely used screening tests for swallowing evaluation is the repetitive saliva swallowing test (RSST). It counts the swallowing reflex and evaluates its inducibility. RSST scores have been reported to correlate with age [6]. Currently, RSST is a safer and easier way to screen for dysphagia, which can evaluate voluntary swallowing. During swallowing, the larynx elevates due to the contraction of several muscles to protect the airway against aspiration. The larynx serves many functions, including swallowing, respiration and vocalization, and its functional impairment leads to an increased incidence of dysphagia or dysphonia and a poor quality of life [7].

Several studies have addressed the efficacy of oral care and exercise in the elderly population. Various treatments have been suggested, including rehabilitation programs, but few have been shown to be as effective as routine singing. Further, studies that investigated the mechanism of action of such agents are rare. Although it has been reported for high-pitched vocal enhancement training [8], no evidence is available regarding the effects of routine singing. Several studies on breath-swallow discoordination [9,10] and magnetoencephalography [11,12] have been performed. Although a previous study investigated the efficacy of singing on slowing the progression of voice aging based on several voice parameters [13], the association between routine singing and voluntary swallowing remains unclear. To our knowledge, no clinical studies have investigated an association between routine vocal training and inducibility of the swallowing reflex. Due to this lack of knowledge, no consensus has been reached as to how routine vocal training should be used in clinical practice. Based on these challenges, the purpose of this study was to understand the impact of the singing experience on inducibility of the swallowing reflex during voluntary swallowing. We hypothesized that singing may have a significant impact on swallowing conditions and performed a cross-sectional study to test this.

## 2. Materials and Methods

### 2.1. Study Design and Oversight

The Institutional Review Board at the National Institute of Information and Communications Technology, Suita, Japan (05 September 2019, Non-Registration Number), Himeji Dokkyo University, Himeji, Japan (13 November 2019, Registration No. 19-13) and Osaka University, Suita, Japan (04 July 2017, Registration No. 16469-2) approved the study protocol. Written informed consent was obtained from all subjects before enrollment. Study procedures were carried out in accordance with the Declaration of Helsinki and the Good Clinical Practice guidelines. The subject’s information was anonymized and de-identified prior to analysis. All anonymized reports; gender, singing experience for at least one year, age, and Body Mass Index (BMI) were reviewed for history associated with impaired swallowing ability. Dysphagia screening was performed using the Japanese version of the 10-item Eating Assessment Tool (EAT-10). A score of 3 or more was defined as indicating a swallowing difficulty [14]. We recorded clinical data along with the Repetitive Saliva-Swallowing Test (RSST), Maximum Phonation Time (MPT) and grip strength scores.

A clinical trial was performed on 212 subjects from Tanba City Cohort in September 2019. The subjects were recruited from the general public with the support of Tanba City. Inclusion criteria were defined as follows: (1) subjects with no history of aspiration pneumonia; (2) no clinically evident cerebrovascular or respiratory disease; (3) no hospitalization within one year, and (4) aged 40 years and over.

**Figure 1:**
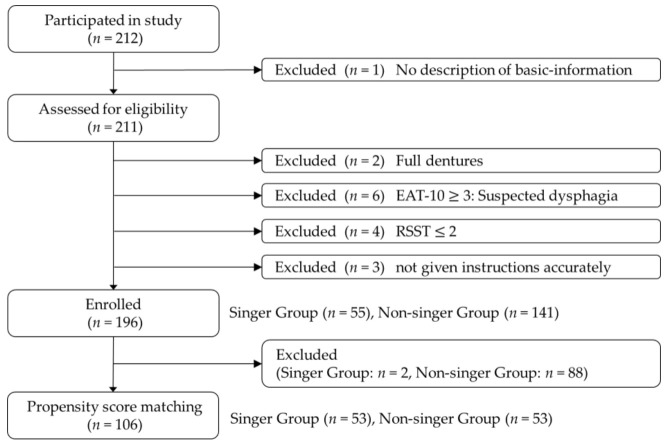
Flow diagram summarizing study selection criteria.

The flow diagram shown in Figure 1 summarizes the study selection criteria. Exclusion criteria were: (1) no basic-information available; (2) full dentures; (3) EAT-10 score >= 3; (4) Repetitive Saliva-Swallowing Test (RSST) score <= 2, and (5) not given accurate instructions. In this cross-sectional study we identified and enrolled 196 subjects (57 males and 139 females) with a mean ± SD age of 60.1 ± 11.7 years old (range 40 to 93 years).

We classified the enrolled 196 subjects into a singer group and a non-singer group based on their singing experience for at least one year. The number of years of singing experience was (mean ± SD) 7.8 ± 10.0 years (range 1 to 50 years). The baseline characteristics of included subjects are shown in Table 1. Of 55 subjects in the singer group, 32 singers were recruited from the general public with the support of Tanba City, and were students taking lessons with Satsuki Adachi, a professional vocalist.

### 2.2. Procedure

Three clinical assessments were performed: RSST, MPT, and grip strength.

#### 2.2.1. RSST

The RSST score measures swallowing function. The speech therapist counts the number of times that saliva is swallowed over thirty seconds. After having the oral cavity moistened with a small amount of water, the subject repeatedly swallows as much as possible in 30 s. The experiment was conducted during the day and a single trial was provided. Less than three swallows indicates dysphagia and is highly correlated with video fluorographic diagnosis with a sensitivity of 0.98 and a specificity of 0.66 [15]. This is one of the most widely used swallowing assessment tests.

#### 2.2.2. MPT

The MPT of aerodynamic inspection measures the longest time in seconds over three attempts that the patient sustains the vowel “a:” as a clinical evaluation of vocal and laryngeal function [16]. The average duration for healthy subjects is 20 s or more, and men can hold it longer. Less than 10 s often interferes with daily conversation. The MPT is affected by general health and lung function [17].

#### 2.2.3. Grip Strength

To assess muscle strength, we recorded grip strength using a hand dynamometer GT-1201D (OG Wellness Technologies Co., Ltd., Okayama, Japan). A single measurement was obtained using the dominant hand while standing.

After subject enrollment, we divided subjects into two groups: singers and non-singers. To ensure the objectivity of the analysis, both singers and non-singers were studied with a large sample size. We then compared differences in clinical outcomes between the subjects with and without singing experience.

### 2.3. Statistical Analysis

Results are expressed as mean ± standard deviation and range for continuous data and frequencies for categorical data. Pearson’s correlation coefficient was used to assess the relationship between each parameter. Comparisons between singers and non-singers were performed using the chi-squared test for categorical variables and two-sided *t*-tests for continuous variables. A *p* value < 0.05 was considered statistically significant. Statistical analyses were performed using JMP Pro 14.2 (SAS Institute Inc., Cary, NC, USA). The assignment of subjects in this cross-sectional study is typically not random. For randomization we matched to ensure that the two groups were similar [18]. Propensity scores assisted with matching variables or covariates. The factors used for matching are described in the Results section. Confirming the matching balance provides an absolute standardized difference [19,20], $asd_{continuous}$ and $asd_{nominal}$.

- The continuous variable covariate case is represented by:
  (1) $$asd_{continuous}=\frac{\left|\bar{x_{singers}}-\bar{x_{\mathrm{non-singers}}}\right|}{\sqrt{\frac{s_{singers}{}^{2}+s_{\mathrm{non-singers}}{}^{2}}{2}}}$$
  where $\bar{x_{singers}}$ is the mean of the singer group, $\bar{x_{\mathrm{non-singers}}}$ is the mean of the non-singer group, $s_{singers}$ is the standard deviation of the singer group and $s_{\mathrm{non-singers}}$ is the standard deviation of the non-singer group.
- The nominal variable covariate case is represented by:
  (2) $$asd_{nominal}=\frac{\left|p_{singers}-p_{\mathrm{non-singers}}\right|}{\sqrt{\frac{p_{singers}\left(1-p_{singers}\right)+p_{\mathrm{non-singers}}\left(1-p_{\mathrm{non-singers}}\right)}{2}}}$$
  where $p_{singers}$ is the rate of the singer group and $p_{\mathrm{non-singers}}$ is the rate of the non-singer group.

To evaluate swallowing ability, univariate analyses between the RSST score and patient characteristics were performed. The RSST scores in the singer group and the non-singer group were compared using an unpaired *t*-test. An analysis of covariance (ANCOVA) revealed a baseline imbalance, and the RSST rank reports underscored data. We performed two ANCOVA analyses. The first analysis included covariates for age, and the second included covariates for grip strength.

## 3. Results

### 3.1. Characteristics of the Subjects

To identify factors that contribute to improved swallowing function we performed a study involving 212 subjects aged 40 years and over. In total, about one-third (*n* = 55) of the 196 subjects were classified as singers having at least one year of singing experience. The EAT-10 screening tool was used to ensure that no subjects had a swallowing disorder. We found a weak correlation between grip strength and age in all classifications (Singers: r = −0.3093, *p* < 0.05, non-singers: r = −0.3150, *p* < 0.0005, Both: r = −0.3196, *p* < 0.0005, Table 2), and between grip strength and BMI (Non-singers: r = 0.3299, *p* < 0.0001).

Of the enrolled 196 subjects, we classified two groups of singers and non-singers based on having at least one year of singing experience. The numbers in each group were unbalanced. Taken together, propensity score matching (PSM) [21,22] was used to adjust for gender, age, BMI and grip strength (Table 3: before matching). A comparison of MPT in groups with and without aspiration has been reported previously, showing MPT in the group with aspiration was significantly shorter in the group without aspiration [23]. It was suggested that a decline of phonatory functions could be a risk factor for aspiration. Therefore, the MPT data were excluded as a matching factor due to their high likelihood of modifying RSST. The criteria used in our matching procedure were based on variables considered to be important determinants and predictors of swallowing ability, which were matched by gender, age, BMI, and grip strength. A 1:1 nearest neighbor within the caliper was defined as the matching allowable area [24], with an initial caliper coefficient of 0.2 and a caliper value of 0.143. Two cases were rejected, while 53 were accepted. The final study population included 106 case-control pairs, with 53 subjects each in the singer group and the non-singer group. Categorical gender comparisons were performed using Pearson’s chi-squared test, while continuous variables (age, BMI and grip strength) were compared using the *t*-test. The *p*-values for gender, age, BMI, and grip strength after matching were larger than those measured before matching. The standardized difference allowed us to identify differences in the range and mean. Statistical significance was measured using an unpaired *t*-test for RSST and MPT between singers and non-singers.

### 3.2. Analysis of Propensity Score Matching

**Figure 2:**
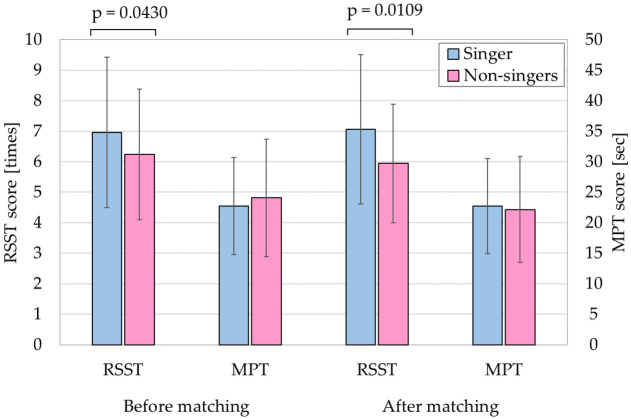
RSST and MPT scores before and after matching.

The RSST score was significantly higher in the singer group than in the non-singer group (singer: 7.1 ± 2.4, *n* = 53 vs. non-singer: 5.9 ± 1.9, *n* = 53, *p* < 0.05, Figure 2). Singers had higher mean RSST scores influenced by having singing experience for at least one year. Table 4 shows the results of univariate analyses in which the dependent variable is the RSST score. No significant correlations were measured in the singer group, while the RSST was significantly associated with age and MPT in the non-singer group (RSST vs. age: *p* = 0.0115, RSST vs. MPT: *p* = 0.0173).

**Figure 3:**
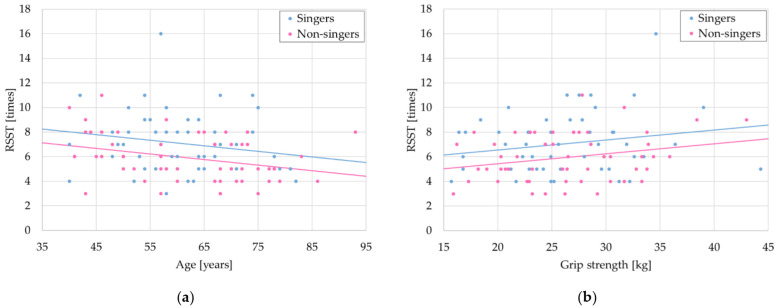
ANCOVA of RSST score: (**a**) ANCOVA of age plotted as a function of RSST score, RSST = −0.045 × Age + 9.837 − 1.122× Group (Singers: Group = 0, Non-singers: Group = 1, *p* = 0.0084); (**b**) ANCOVA of grip strength plotted as a function of RSST score, RSST = 0.081 × Grip strength + 4.931 − 1.124 × Group (Singers: Group = 0, Non-singers: Group = 1, *p* = 0.0089).

The above results can be attributed to baseline imbalance. ANCOVA adjusted for age and grip strength on RSST yielded a statistically significant *p*-value of 0.01 (Figure 3).

## 4. Discussion

This is the first report, to our knowledge, that found that singers have better inducibility of swallowing reflex than non-singers. Of particular note is that singing experience was associated with an increased rate of inducibility of the swallowing reflex. Our findings underscore the fact that vocal training may also affect intervention efficiency. In this study we showed that singing experience affected RSST score and that singers are statistically superior in their inducibility of the swallowing reflex. Regarding RSST scores, it was reported previously that normal values of RSST based on RSST score were 7.4 for normal young subjects and 5.9 for normal elderly subjects [6]. In this study, we were targeted healthy singers and non-singers. Though the results were higher than the screening value of RSST, similar values to previous studies suggested that they were in the normal range of the values of healthy subjects. We conducted a cross-sectional study of singers and non-singers to gain insight into the clinical significance of minimal functional deterioration of singers due to aging. Our results support the hypothesis that singing experience is associated with swallowing ability, and that vocal training interventions focused on improved inducibility of the swallowing reflex will be better for the treatment and rehabilitation of dysphagia. Understanding the changes in inducibility of the swallowing reflex that accompanies age is of critical importance. Our results suggest that vocal training decreased the risk of developing dysphagia over the course of aging. The elderly population would, therefore, be better served by vocal treatment.

### 4.1. Comparison between Singers and Non-Singers

All the subjects in the singer group and non-singer group were recruited and enrolled with inclusion and exclusion criteria to prevent selection bias. Both groups had similar health conditions and oral conditions. Inclusion criteria included determinations based on not having full dentures, swallowing conditions determined by EAT-10 and RSST scores, and cognitive conditions associated with understanding instructions accurately. The mean and range data for singers and non-singers implied similar health conditions of the enrolled subjects before matching in Table 3A. We report negative correlations between age and RSST, grip strength, and MPT on singers and non-singers in Table 2, indicating age-based tendencies were the same in each group. Interestingly, the negative correlation coefficient between age and RSST after matching, as shown in Table 4, was higher than that before matching, as shown in Table 2, in the non-singers group. In addition, propensity score matching slightly increased the mean difference and significance on RSST between the two groups (Figure 2). We therefore suggest that matching eliminated the error due to spread of the distribution. A shown in Figure 3, to investigate whether singing experience for at least one year affects RSST, we performed ANCOVA with the covariates of age and grip strength, respectively. We set a covariate of age, which is closely related to RSST, and a covariate of grip strength, which is weak correlated with age. The scores for singers were higher than those for non-singers, indicating that singing experience influences RSST scores. In our previous study [25], we performed a preliminary trial to evaluate laryngeal movement by applying deep-learning to a video of swallowing. This confirmed that the period of a single swallowing event for singers was significantly shorter than that for non-singers. This should contribute to the increase in RSST scores of singers.

### 4.2. Dysphagia Treatment of Swallowing Therapy

Many studies and case reports have evaluated swallowing training practice related to feeding and basic exercise. However, few studies present credible evidence regarding the usefulness of these treatments [8,26]. A systematic review in collaboration with the American Speech-Language-Hearing Association and the Department of Veterans Affairs summarized how to incorporate Evidence-based Medicine (EBP) into clinical studies by providing an overview of swallowing posture and voluntary swallowing [27]. Although this work reported that compensatory methods had statistically significant beneficial effects, the management of exclusion bias remains a major challenge. The elderly responded to a head-raising exercise, resulting in augmentation of a deglutitive upper esophageal sphincter (UES) opening [28,29], but the outcome of this work could not exclude the influence of variability attributable to individual training effort. It has been previously shown that a decline in voice stability is more frequently observed in non-singers compared with singers, and that singing effects are associated with aging [7]. Vocalization supports the respiratory musculature, including pharyngeal and laryngeal units. It is therefore conceivable that vocalization increases the influence of swallowing movements by using the same muscles as vocalization and by elevating the larynx. This may have great promise in the treatment of internal laryngeal muscles naturally used during singing [13].

### 4.3. Study Limitation

The sample size in our study was relatively small, especially of male subjects. Our findings should be interpreted with caution due to uncertainties around some of the model parameters and baseline data. There is a need for further studies to test our findings in a larger population. While the physiological mechanism of vocal training needs to be explained, it is important to note that it offers many clinical benefits. Further research is needed to investigate the relationship between vocal training and brain activity [30] and understand the mechanisms behind swallowing function and disorder.

## 5. Conclusions

In summary, we found a difference in inducibility of the swallowing reflex between singers and non-singers. Our findings provide evidence that vocal training may be correlated with improved swallowing regulation related to inducibility of the swallowing reflex. To our knowledge, this is the first study to demonstrate the impact of routine vocal training on inducibility of the swallowing reflex.

## Figures and Tables

**Table 1:** Full Cohort Demographic and Clinical Data.

		All Subjects	
N		196	
		Male	Female
Group	Singers	6	49
	Non-singers	51	90
		Mean ± SD	range
Age	[years]	60.1 ± 11.7	40–93
BMI	[kg/m^2^]	22.4 ± 3.2	16.2–38.8
Grip strength	[kg]	29.9 ± 8.9	15.7–55.3
RSST	[times]	6.4 ± 2.2	3–16
MPT	[sec]	23.7 ± 9.2	6–62

Continuous variables are presented as the mean ± SD and range.

**Table 2:** Correlations between parameters associated with swallowing ability.

		RSST	Age	BMI	Grip Strength	MPT
Singers	RSST	1.0000	−0.1815	−0.2037	0.2219	0.2605
	Age	−0.1815	1.0000	0.2306	−0.3093 *	−0.0192
	BMI	−0.2037	0.2306	1.0000	0.1317	−0.1448
	Grip strength	0.2219	−0.3093 *	0.1317	1.0000	0.2402
	MPT	0.2605	−0.0192	−0.1448	0.2402	1.0000
Non-singers	RSST	1.0000	−0.2344 **	−0.0335	0.2446 ^#^	0.1778 *
	Age	−0.2344 **	1.0000	−0.1096	−0.3150 ^##^	−0.2043 *
	BMI	−0.0335	−0.1096	1.0000	0.3299 ^##^	−0.0284
	Grip strength	0.2446 ^#^	−0.3150 ^##^	0.3299 ^##^	1.0000	0.2829 ^##^
	MPT	0.1778 *	−0.2043 *	−0.0284	0.2829 ^##^	1.0000
Both	RSST	1.0000	−0.2042 ^#^	−0.0884	0.1815 *	0.1859 **
	Age	−0.2042 ^#^	1.0000	−0.0382	−0.3196 ^##^	−0.1696 *
	BMI	−0.0884	−0.0382	1.0000	0.2965 ^##^	−0.0501
	Grip strength	0.1815 *	−0.3196 ^##^	0.2965 ^##^	1.0000	0.2811 ^##^
	MPT	0.1859 **	−0.1696 *	−0.0501	0.2811 ^##^	1.0000

* *p* < 0.05, ** *p* < 0.01, ^#^ *p* < 0.005, ^##^ *p* < 0.001.

**Table 3:** Covariate imbalance prior to matching and matched samples.

(**A**): Before matching						
**Matching** **Factor**			**Singers**	**Non-Singers**	***p*-Value**	**Standardized**
	N		55	141		
Used	Gender				0.0005	0.6237
	Male	([%])	6 (10.9)	51 (36.2)		
	Female	([%])	49 (89.1)	90 (63.8)		
	Age	[years]	61.5 ± 10.2	59.6 ± 12.2	0.3039	0.1704
	(range)		(40–82)	(40–93)		
	BMI	[kg/m^2^]	22.2 ± 2.9	22.6 ± 3.3	0.4302	0.1289
	(range)		(16.9–29.8)	(16.2–38.8)		
	Grip Strength	[kg]	26.0 ± 5.8	31.4 ± 9.5	0.0001	0.6843
	(range)		(15.7–44.3)	(15.9–55.3)		
Non-used	RSST	[times]	7.0 ± 2.5	6.2 ± 2.1	0.0430	0.3134
	(range)		(3–16)	(3–13)		
	MPT	[sec]	22.7 ± 7.9	24.1 ± 9.7	0.3651	0.1504
	(range)		(8–45)	(6–62)		
(**B**): After matching						
**Matching** **Factor**			**Singers**	**Non-Singers**	***p*-Value**	**Standardized**
	N		53	53		
Used	Gender				1.0000	0.0000
	Male	([%])	5 (9.4)	5 (9.4)		
	Female	([%])	48 (90.6)	48 (90.6)		
	Age	[years]	61.4 ± 10.3	61.2 ± 13.5	0.9292	0.0173
	(range)		(40–82)	(40–93)		
	BMI	[kg/m^2^]	22.1 ± 3.0	22.5 ± 3.6	0.5175	0.1262
	(range)		(16.9–29.8)	(16.8–38.8)		
	Grip Strength	[kg]	26.2 ± 5.8	26.4 ± 5.9	0.9149	0.0208
	(range)		(15.7–44.3)	(15.9–43.0)		
Non-used	RSST	[times]	7.1 ± 2.4	5.9 ± 1.9	0.0109	0.5039
	(range)		(3–16)	(3–11)		
	MPT	[sec]	22.7 ± 7.8	22.1 ± 8.7	0.7162	0.0708
	(range)		(8–45)	(10–52)		

Continuous variables are presented as the mean ± SD (range).

**Table 4:** Univariate analysis of RSST score.

	Singers			Non-Singers		
Factor	r	95% CI	*p*-Value	r	95% CI	*p*-Value
Age	−0.1590	−0.4116–0.1163	0.2554	−0.3447	−0.5626–−0.0821	0.0115
BMI	−0.1930	−0.4404–0.0815	0.1661	−0.1442	−0.3989–0.1312	0.3030
Grip strength	0.1859	−0.0889–0.4344	0.1826	0.2576	−0.0137–0.4935	0.0626
MPT	0.2458	−0.0263–0.4839	0.0761	0.3258	0.0609–0.5478	0.0173

r: Pearson’s correlation coefficient.
